# Mechanisms of arterial remodeling: lessons from genetic diseases

**DOI:** 10.3389/fgene.2012.00290

**Published:** 2012-12-13

**Authors:** Bernard J. van Varik, Roger J. M. W. Rennenberg, Chris P. Reutelingsperger, Abraham A. Kroon, Peter W. de Leeuw, Leon J. Schurgers

**Affiliations:** ^1^Department of Internal Medicine, Medical Centre and Cardiovascular Research Institute Maastricht, Maastricht UniversityMaastricht, Netherlands; ^2^Department of Biochemistry, Cardiovascular Research Institute Maastricht, Maastricht UniversityMaastricht, Netherlands

**Keywords:** arterial remodeling, calcification, genetic disease, vitamin K, vitamin K-antagonists

## Abstract

Vascular disease is still the leading cause of morbidity and mortality in the Western world, and the primary cause of myocardial infarction, stroke, and ischemia. The biology of vascular disease is complex and still poorly understood in terms of causes and consequences. Vascular function is determined by structural and functional properties of the arterial vascular wall. Arterial stiffness, that is a pathological alteration of the vascular wall, ultimately results in target-organ damage and increased mortality. Arterial remodeling is accelerated under conditions that adversely affect the balance between arterial function and structure such as hypertension, atherosclerosis, diabetes mellitus, chronic kidney disease, inflammatory disease, lifestyle aspects (smoking), drugs (vitamin K antagonists), and genetic abnormalities [e.g., pseudoxanthoma elasticum (PXE), Marfan's disease]. The aim of this review is to provide an overview of the complex mechanisms and different factors that underlie arterial remodeling, learning from single gene defect diseases like PXE, and PXE-like, Marfan's disease and Keutel syndrome in vascular remodeling.

## Introduction

Arterial remodeling refers to the myriad of structural and functional changes of the vascular wall that occur in response to disease, injury, or aging. Although arterial remodeling can be regarded as a mechanism that naturally occurs with aging, early arterial remodeling is associated with significant hemodynamic changes and cardiovascular morbidity and mortality. Arterial remodeling is set into motion by a variety of complex pathophysiological mechanisms that are closely interrelated, and that influence both the cellular and non-cellular components of the vascular wall. Mechanisms involved in arterial remodeling include fibrosis, hyperplasia of the arterial intima and media, changes in vascular collagen and elastin, endothelial dysfunction, and arterial calcification. Migration and proliferation of vascular smooth muscle cells (VSMCs) contribute to thickening of the arterial intima. Differentiation of VSMCs from their contractile to a secretory or osteogenic phenotype may lead to increased vascular tone, and promotes extracellular matrix (ECM) calcification. Additionally, alterations in the activity of vitamin K-dependent proteins may affect the progression of vascular remodeling, including the induction of calcification. Because of this complexity, it is difficult to study to what extent a single mechanism contributes to arterial remodeling. Monogenetic diseases such as pseudoxanthoma elasticum (PXE), PXE-like syndrome, Marfan's syndrome or Keutel syndrome are characterized by a clinical phenotype that is similar to that of arterial remodeling, but are caused by a specific defect that affects only one or several pathophysiological mechanisms of arterial remodeling. Lessons learned from these relatively rare diseases may therefore ultimately provide insight in more common, multifactorial cardiovascular diseases such as hypertension, diabetes mellitus, and chronic kidney disease as well as in normal vascular aging.

## General features of arterial remodeling

**Figure 1:**
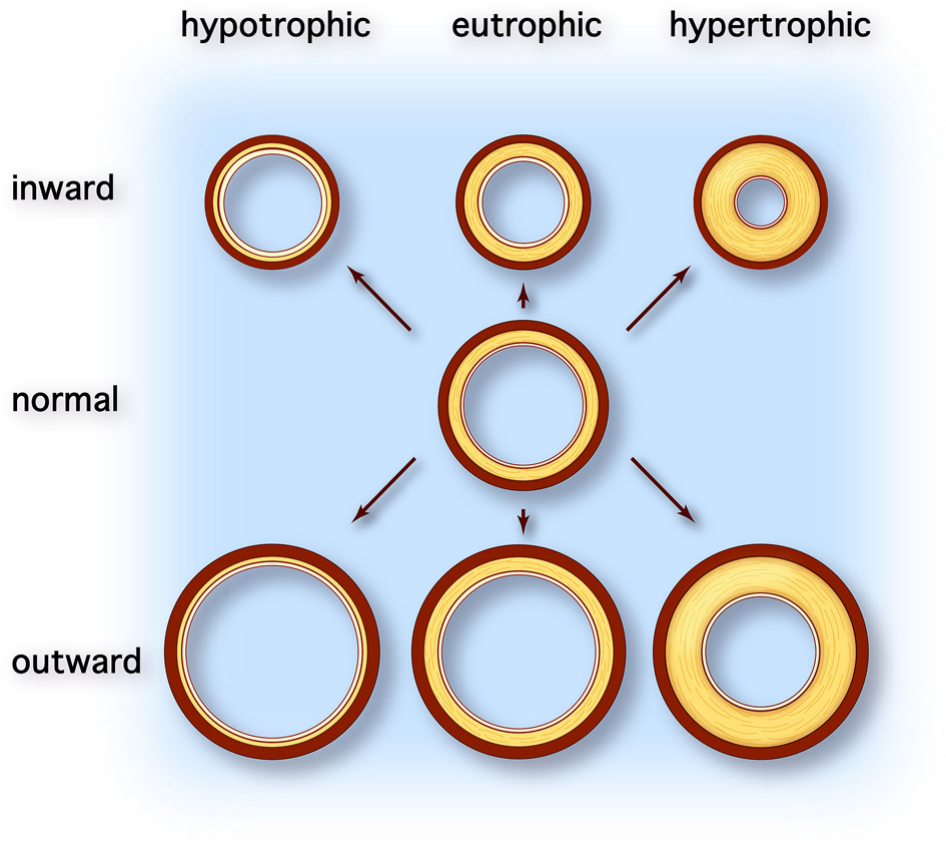
**Types of vascular remodeling.** Adapted from Mulvany et al. (1996). Different types of arterial remodeling can be distinguished: hypotrophic (left column), eutrophic (center column) and hypertrophic (right column). In addition remodeling can be either inward or outward. Hypotrophic remodeling results in a relative thinner wall and a lower wall-to-lumen ratio. Conversely hypertrophic remodeling is characterized by thickening of the vascular wall due to cellular hyperplasia and/or hypertrophy or deposition of extracellular matrix material and results in increased wall-to-lumen ratio. When the diameter of the vessel changes but the wall-to-lumen ratio remains the same it is called eutrophic remodeling. All types of arterial remodeling can occur in cardiovascular disease, depending on the underlying pathophysiology (e.g., aneurysm or hypertensive arterial stiffening) and arterial site (e.g., central elastic arteries vs. peripheral resistance arteries).

Arterial remodeling is thought to reflect adaptation of the vessel wall to mechanical and hemodynamic stimuli (Nichols and O'Rourke, 2005). Arterial remodeling is characterized by alterations in the structure and function of the vascular wall and can be divided into atherosclerosis and arteriosclerosis. Whereas atherosclerosis is characterized by a focal inflammatory process in the intima initiated by accumulation of lipids in plaques, arteriosclerosis is a more diffusely localized alteration of the medial arterial vascular wall (Libby, 2002). Arteriosclerosis is associated with aging and generalized cardiovascular, metabolic, or inflammatory disease. Macroscopically, different types of arterial remodeling can be distinguished, depending on the type and localization of the vessel (Figure 1) (Mulvany et al., 1996). Arterial remodeling can be either inward or outward and can be hypertrophic (thickening of the vascular wall), eutrophic (constant wall thickness), or hypotrophic (thinning of the vascular wall) (Mulvany et al., 1996). Changes observed in arteriosclerotic arterial remodeling are mainly seen in large central elastic arteries. They are characterized by increased vessel diameter and thickened intimal and medial layers of the vascular wall (outward hypertrophic remodeling) (O'Rourke and Hashimoto, 2007). On the other hand, remodeling of muscular peripheral vessels is more often inwardly eutrophic or hypertrophic, probably reflecting sustained vasoconstriction of vessels (Mulvany, 2008).

Thickening of the arterial wall is caused by intimal hyperplasia, medial hypertrophy and hyperplasia of VSMCs, and deposition of ECM material including minerals (Virmani et al., 1991; Safar et al., 1998; Schwartz et al., 2000). The normal composition and lay-out of ECM of the vascular wall is disrupted in arterial remodeling. In the media of the normal arterial wall, elastic fibers are arranged in parallel, concentric, fenestrated layers, alternating with layers of VSMCs anchored to the elastic fibers and structural fibers by glycoproteins and integrins (Dingemans et al., 2000; Nichols and O'Rourke, 2005). These structures, termed elastic lamellae, enable the vessel to expand and buffer the systolic blood pressure pulse, while simultaneously maintaining structural stability. Elastic fibers provide passive elastic buffering, whereas VSMCs dynamically redistribute tensile stress across fibers due to their ability to contract and relax (Rachev and Hayashi, 1999). With arterial remodeling the layered architecture of elastic lamellae is lost as they become progressively fragmented and fibrotic (Farand et al., 2007). At higher levels of blood pressure, vessels dilate which results in increased tensile stress on the vascular wall, in accordance with LaPlace's Law of circumferential wall tension (Nichols and O'Rourke, 2005). Thickening of the arterial wall occurring with arterial remodeling reduces tensile stress. VSMCs of adults do not synthesize new elastin but mainly non-elastic collagen resulting in stiffening of the vascular wall (Greenwald, 2007). Closely related to the degradation of ECM, the deposition of calcium minerals further contributes to stiffening and remodeling of vascular tissue (Blaha et al., 2009; Sekikawa et al., 2012).

In addition to structural changes, endothelial function plays an important role in arterial remodeling. Blood flow and shear stress stimulate endothelial cells to produce nitric oxide (NO), which in turn influences contraction and relaxation of VSMCs. Endothelial function decreases with age and endothelial dysfunction is common in many cardiovascular diseases. Moreover, in response to pathological conditions, such as altered shear stress or inflammation, endothelial cells produce cytokines and growth factors that influence the homeostasis of the vascular wall (Csiszar et al., 2009; Urschel et al., 2012). Endothelial cells produce transforming growth factor-beta (TGF-β) and bone morphogenetic proteins (BMPs) which stimulate VSMCs and vascular pericytes to proliferate, to differentiate and to deposit ECM matrix (discussed in more detail below) (Simionescu et al., 2005; Boström et al., 2011).

## Pathogenesis of arterial remodeling

**Figure 2:**
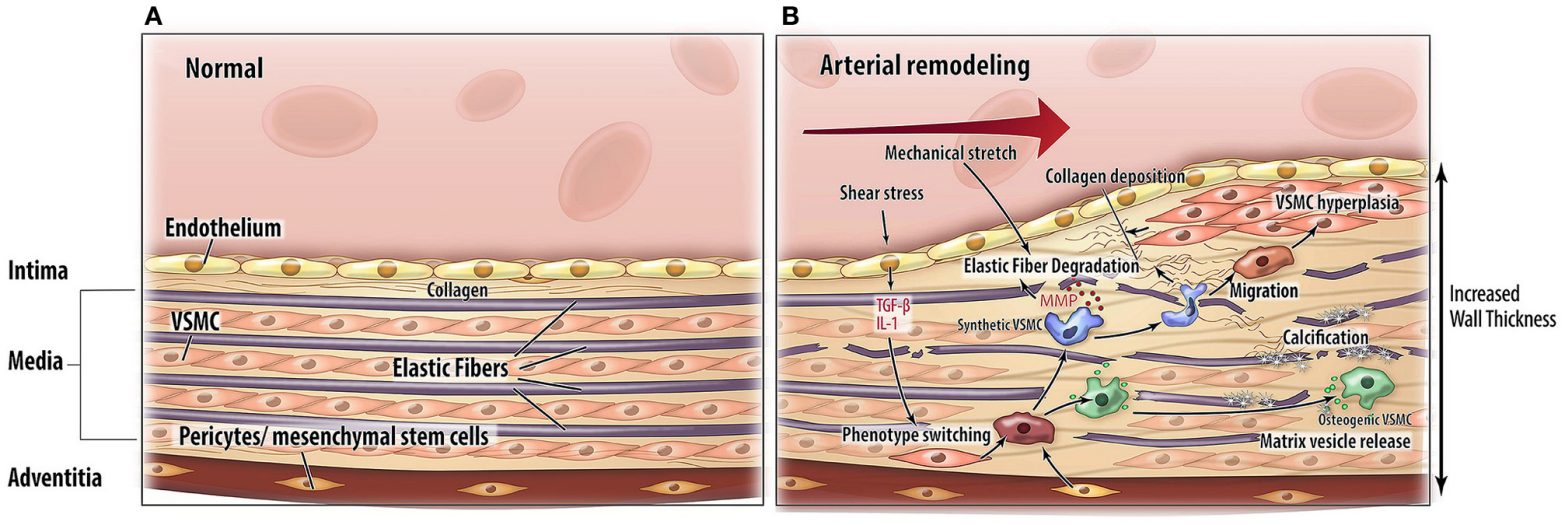
**Pathophysiological mechanisms of arterial remodeling.** Cross sectional schematic view of the arterial wall. **(A)** Normal situation. **(B)** Arterial remodeling. Arterial remodeling is characterized by thickening of the wall. Elastic fiber degradation, extracellular matrix calcification and collagen deposition lead to adaptation of the vascular wall. Abbreviations: TGF-β, transforming growth factor-beta; IL-1, interleukin 1; MMP, matrix metalloproteinases; VSMC, vascular smooth muscle cell.

**Figure 3:**
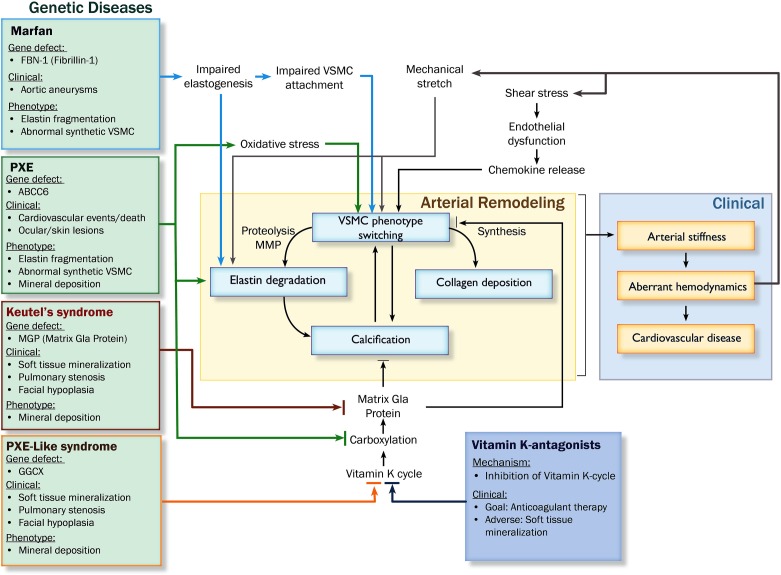
**Pathophysiological pathways leading to arterial remodeling in genetic and cardiovascular disease.** Abbreviations: PXE, pseudoxanthoma elasticum; MMP, matrix metalloproteinases; VSMC, vascular smooth muscle cell; GGCX, gamma glutamyl transferase.

Arterial remodeling is driven by numerous, highly regulated and interrelated processes. Processes that are of particular importance as they are central in arterial remodeling include: (1) VSMC proliferation and differentiation, (2) degradation and fracture of elastin fibers, and (3) calcification and deposition of ECM material (Figure 2). Genetic diseases with a phenotype resembling vascular disease all affect one or several of these key processes and may thus provide more insight in the mechanisms of vascular disease (Figure 3).

### Vascular smooth muscle cell proliferation and differentiation

VSMCs are key regulators of vascular tone and health and insight into their function is of utmost importance for our understanding of the causes of arterial remodeling. In normal arteries, VSMCs in the tunica media regulate vessel tone and diameter in order to maintain hemodynamic balance (Alexander and Owens, 2012). To fulfill this regulatory function, VSMCs need to have a contractile phenotype. Contractile VSMCs are characterized by a number of phenotype-specific marker proteins such as smooth muscle 22-alpha (SM22α), alpha-smooth muscle actin (αSMa), and smoothelin (Iyemere et al., 2006; Eys et al., 2007). Although the majority of VSMCs in the vascular wall display a contractile phenotype, studies have shown that a specific subset of medial VSMCs has the ability to differentiate into a synthetic phenotype which can be further subdivided into a migratory-proliferative phenotype, a secretory phenotype or an osteogenic phenotype (Gerthoffer, 2007). Phenotypic flexibility of VSMCs is necessary to deal with the varying conditions of vascular tissue. Stress signals switch gene expression that will modulate VSMC phenotype to adapt. This process of differentiation is termed phenotype switching and is considered to be a key mechanism in arterial remodeling (Iyemere et al., 2006; Alexander and Owens, 2012).

Phenotype switching occurs in response to vascular injury or stress and is characterized by reduced expression of genes which are specific for contractile VSMCs and cellular morphology (Alexander and Owens, 2012). Although the precise mechanisms are still not fully understood, many different stimuli have been identified, some of which are summarized in Table 1 (Alexander and Owens, 2012). Migratory stimuli, for instance, alter the cytoskeleton of VSMCs. As a consequence, cell adhesion molecules are detached from the ECM and surrounding vascular cells. Lamellipodia protrude from the leading edge of the cell due to actin polymerization, enabling it to move through the ECM toward a chemotactic stimulus (Willis et al., 2004). This migration contributes to intimal VSMC proliferation and hyperplasia, which is an important cause of arterial wall thickening.

**Table 1:** **Stimuli for vascular smooth muscle phenotype switching**.

Inflammation
Oxidative stress
Hemodynamic shear stress
Mechanical stretch
Advanced glycation end products (AGE)
Increased calcium-phosphate product
**SYSTEMIC HORMONAL**
Angiotensin II (Ang II)
Aldosterone
**PARACRINE STIMULI**
Transforming growth factor-β (TGF-β)
Fibroblast growth factor (FGF)
Endothelial growth factor (EGF)
Platelet derived growth factor (PDGF)
Matrix metalloproteinases (MMP)

Synthetic VSMCs produce elastolytic enzymes (matrix metalloproteinases; MMPs), which facilitate migration by detaching cells from the basement membrane and ECM. Indeed, upregulation of MMPs coincides with the migration of VSMCs (Willis et al., 2004). A genetic disorder that is associated with VSMC phenotype switching is Marfan's disease. It is characterized by abnormal synthesis and function of elastic fibers (Kielty, 2006). Patients with Marfan's disease suffer from abnormal growth, skeletal disorders, ocular problems and increased tendency to develop aneurysms. The gene defect underlying Marfan's disease is a mutation of the fibrillin-1 (FBN-1) gene, which encodes the glycoprotein FBN-1. FBN-1 is essential for maintaining structural stability of elastic fibers, as well as attaching VSMCs to the elastic fibers (Bunton et al., 2001). Because of defective synthesis, elastic fibers are prone to early mechanical fragmentation and therefore disruption of elastic laminae. However, additional studies on the pathophysiological mechanisms in Marfan's disease showed that, preceding elastic fiber degradation, impaired binding of VSMCs-induced differentiation into a synthetic proteolytic phenotype (Galis et al., 1994; Bunton et al., 2001; Galis and Khatri, 2002). The resulting production of MMPs damages the already weakened vascular wall (Pratt and Curci, 2010). These patho-mechanistic changes in Marfan's disease help to understand underlying mechanisms leading to general vascular disease. Indeed, Goodall et al. showed that VSMCs from inferior mesenteric veins of patients with aortic aneurysms display increased MMP-2 production and an increased number of migratory VSMCs (Goodall et al., 2002). Bendeck et al. demonstrated that inhibition of MMP activity inhibited VSMC migration in rats (Bendeck et al., 1996). Moreover, VSMCs are important for atherosclerotic plaque stability. VSMCs and myofibroblasts in the fibrous cap provide stability to atherosclerotic plaques if they deposit collagen. On the contrary, if a significant part of these VSMCs display a proteolytic phenotype, degradation of fibrous cap material may facilitate plaque rupture (Johnson, 2007). Therefore, the role of VSMCs in maintaining atherosclerotic plaque stability largely depends on VSMC phenotype, stressing out the importance to find therapeutic agents that are able to modify the VSMC phenotype (Orr et al., 2010).

#### Osteogenic VSMC phenotype

Under specific stimuli such as sustained high extracellular levels of calcium and phosphate or in the absence of inhibitors of calcification, VSMCs can differentiate into an osteogenic phenotype in which VSMCs acquire features usually observed in chondrocytes and osteoblasts (Shanahan et al., 1994; Iyemere et al., 2006). Osteogenic VSMCs are characterized by down regulation of mineralization inhibitory proteins, upregulation of alkaline phosphatase and release of matrix vesicles (MVs) (Shanahan et al., 2011). *In vitro*, culturing VSMCs with elevated phosphate concentrations results in up-regulation of osteogenic markers (Runx2, osterix, and alkaline phosphatase) and down-regulation of VSMC lineage markers (SMa actin, SM22a) (Shanahan et al., 2011). Downstream, bone morphogenetic protein-2 (BMP-2) induces an osteogenic differentiation of VSMCs. BMP-2 has been shown to be expressed in human atherosclerotic lesions (Boström et al., 1993). The phenotypic switch of VSMCs to chondrocyte- and osteoblast-like cells by BMP-2 is limited by calcification inhibitory proteins such as matrix Gla-protein (MGP). In MGP knock-out mice, the absence of MGP results in heavily calcified elastic fibers, and loss of VSMCs which are differentiated into chondrocytic VSMCs (Luo et al., 1997). Additionally, MGP deficiency in VSMCs results in decreased smooth muscle markers which is accompanied by an up-regulated expression of the bone-specific transcription factor cbf1a/Runx2 and the osteogenic protein osteopontin (Speer et al., 2002). The ability of MGP to keep VSMCs in the contractile phenotype may be accomplished by binding BMP-2 (Wallin et al., 2000; Zebboudj et al., 2003).

Tanimura and co-workers were the first to report an association between small membrane encapsulated particles, MVs, and vascular calcification (Tanimura et al., 1983). Vesicular structures have been found in both intimal and medial layers and were likely derived from VSMCs (Kim, 1976; Bennett et al., 1995; Hsu and Camacho, 1999). The release of vesicle bodies from VSMCs was first described as a rescue mechanism against calcium overload trying to prevent apoptosis of VSMCs (Fleckenstein-Grün et al., 1992). VSMC-derived MVs have been identified in human arteries in association with atherosclerosis and hypertension (Kim, 1976; Kockx et al., 1998). *In vitro*, MV from VSMCs form the nidus for calcification (Shanahan et al., 1999).

### Degradation and fracture of elastin fibers

#### Elastin

Elastic fibers consist of polymers of tropoelastin cross-linked to fibrillin-rich microfibrils. In the vasculature, elastin is mainly produced during the fetal and neonatal period by (secretory) VSMCs. Above we discussed the importance of elastin for maintaining arterial wall stability and VSMC homeostasis in Marfan's Disease. Additionally, elastin is also an important nidus for calcification. This is illustrated in PXE disease and its accompanying clinical features. PXE is characterized by extensive calcification that mainly occurs along elastic fibers. Although cutaneous manifestations are primarily of cosmetic concern, presence of characteristic skin lesions signifies risk for development of vascular calcification with considerable morbidity and occasional early mortality (Uitto et al., 2010).

Even in the absence of diseases which directly affect elastin structure and function, similar processes can be observed in vascular aging and aortic stiffening (Smith et al., 2012). The question remains, what causes disruption of elastic fibers associated with aging? Initially, it was hypothesized that elastin degradation was predominantly the result of material fatigue caused by cyclic stretching of elastic fibers with every heart beat (O'Rourke, 1976; Nichols and O'Rourke, 2005). Diseases such as (systolic) hypertension would accelerate this process, since increased pulse pressure (PP) exerts greater tensile stress on the vascular wall and increased stretch on fibers. In support of this hypothesis, structural alterations in elastin have been demonstrated to be inversely associated with total number of heart beat cycles *in vitro* (Avolio et al., 1998). However, there are no *in vivo* studies supporting mechanical fragmentation of elastin.

### Calcification and deposition of ECM material

Both VSMC phenotype switching and ECM degradation result in enhanced and accelerated vascular calcification. Initially, vascular calcification was regarded as passive mineral deposition. However, this view has been abandoned since overwhelming evidence exists that vascular calcification actually is a highly regulated process. Soft tissue calcification is thought to result from an imbalance between calcification-promoting and -inhibiting factors (Table 2). Calcification is the hallmark of patients with genetic diseases like Keutel's syndrome, PXE, and PXE-like syndrome (Ziereisen et al., 1993; Munroe et al., 1999; Vanakker et al., 2007; Rutsch et al., 2011). Keutel's syndrome is caused by a mutation in the gene encoding MGP, which is considered to be the most important inhibitor of vascular calcification. MGP is a 14 kD protein which requires vitamin K-dependent carboxylation to become biologically active. Clinically, lessons learned from the mechanisms underlying Keutel's disease can help understanding vitamin K-antagonist-induced vascular calcifications (discussed below) (Rennenberg et al., 2010; Weijs et al., 2011; Schurgers et al., 2012).

**Table 2:** **Calcification regulating factors**.

**FACTORS PROMOTING CALCIFICATION**
Bone morphogenetic protein 2 (BMP-2)
↑ Calcium-phosphate product
Tumor Necrosis Factor α (TNF-α)
Interleukin 6 (IL-6)
Receptor activator of nuclear factor κB (RANK) ligand (RANKL)
Insulin-like growth factor I (IGF-I)
Insulin
↑ Glucose
↑ Parathyroid hormone
Matrix metalloproteinases (MMP)
Elastin degradation
Hydroxyapatite crystals
**FACTORS INHIBITING CALCIFICATION**
Fetuin-A
Matrix gla protein (MGP)
Osteoprotegerin (OPG)

In PXE, the underlying genetic defect is a loss-of-function mutation of the abcc6 gene. This gene encodes a transmembrane transporter protein (Multi Drug Resistant Protein 6; MDRP-6). The substrate of the MDRP-6 is not known, and the exact mechanisms by which this mutation leads to elastin calcification are not yet fully understood. Recent studies have pointed toward calcification being stimulated by phenotype switching of VSMCs, oxidative stress, and interference with carboxylation of MGP (Pasquali-Ronchetti et al., 2006; Garcia-Fernandez et al., 2008; Boraldi et al., 2009; Li et al., 2009b; Rutsch et al., 2011). Similarly, in PXE-like syndrome a mutation in the γ-glutamylcarboxylase (GGCX) gene causes elastic fiber calcification as is observed in vitamin K-antagonist-induced vascular calcification (Gheduzzi et al., 2007; Vanakker et al., 2007; Rennenberg et al., 2010; Weijs et al., 2011; Schurgers et al., 2012). The GGCX mutation is associated with increased bleeding tendency due to impairment of vitamin K-dependent coagulation factors (Vanakker et al., 2007; Li et al., 2009a). This has led to the concept that vitamin K-dependent proteins are of importance in inhibiting vascular elastin calcification. The GGCX mutation results in decreased activity of MGP and subsequently an impaired inhibitory potential for calcification, similar to the situation in Keutel's syndrome in which MGP is absent (Schurgers et al., 2008; Vanakker et al., 2010). In a similar manner, treatment with vitamin K-antagonists may also induce an increased tendency for calcification (Figure 2) (Price et al., 1998; Schurgers et al., 2007; Rennenberg et al., 2010; Chatrou et al., 2012). Since vitamin K-antagonists work by inhibiting the Vitamin K cycle and by reducing carboxylation of MGP, these findings confirm the important central role of MGP in the regulation of calcification. Therefore, it is highly probable that in these diseases, MGP also plays an important regulatory role in calcification (Shanahan et al., 1999; Schurgers et al., 2007).

## Clinical aspects of arterial remodeling

Since the normal function of vessels is to maintain adequate perfusion of organs and tissues and to buffer oscillating blood pressures, arterial remodeling results in changes in this function. At first, these are compensatory (i.e., reducing wall tension). However, in later stages these compensatory mechanisms become detrimental and initiate a vicious cycle of pathophysiological aberrations.

### Arterial remodeling, arterial stiffness and damaging hemodynamics

**Figure 4:**
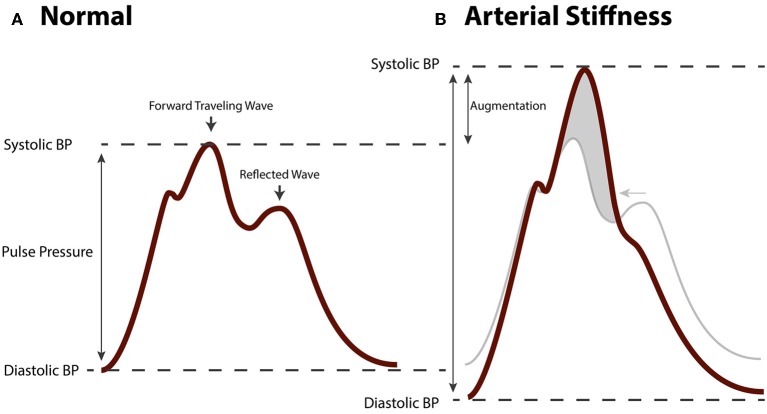
**Hemodynamic changes in arterial stiffening. (A)** Aortic blood pressure waveform of a healthy, normotensive person. The forwards traveling wave precedes the (backwards traveling) reflected wave. **(B)** Aortic pressure waveform of a person with arterial stiffness. Due to increased pulse wave velocity, the forward traveling wave and reflected wave are summated leading to augmented pulse pressure.

Fragmentation of the elastic lamina, hyperplasia and hypertrophy of VSMC, loss of contractility of VSMC, deposition of collagen, and arterial calcification lead to stiffening of arteries. Many studies have shown that arterial stiffness, which is clinically measured as the carotid-femoral pulse wave velocity (cfPWV), is independently associated with cardiovascular risk and mortality (Laurent et al., 2001, 2012; Mitchell et al., 2010; Vlachopoulos et al., 2010). In addition, arterial stiffness is independently associated with, and predictive of target organ damage of the heart, kidneys, and brain (Laurent and Boutouyrie, 2005). Arterial stiffness reflects the degree of remodeling in large arteries and is used as a parameter for cardiovascular risk stratification next to traditional cardiovascular risk factors (Nurnberger et al., 2002). The mechanism linking arterial stiffness to an adverse outcome is thought to involve a pathological hemodynamic profile in large, central arteries such as the aorta (Mitchell, 2009). This pathological hemodynamic pattern consists of an increased systolic blood pressure (SBP; i.e., systolic hypertension) and decreased diastolic blood pressure (DBP) resulting in an increased PP. The pressure waveform in the aorta is composed of a forward traveling wave generated by contraction of the left ventricle of the heart, and a backwards traveling wave generated by reflection from peripheral arteries (Figure 4A). This reflected wave is generated at vascular bifurcations and at sites where the elastic conduit arteries transition into muscular resistance arteries (Mitchell, 2004). At this site the difference in impedance of the vascular wall causes the forward traveling wave to be reflected. The shape of the aortic pressure waveform is largely determined by timing and speed with which the pulse wave propagates through the arteries. With arterial stiffening the speed of both the forward and backward traveling wave is increased. Remodeling of arteries causes an earlier wave reflection. As a result of different timing of both waves, the forward traveling wave and the reflected wave are summated, leading to an augmented systolic peak and a relatively low DBP (Figure 4B), generating a highly pulsatile flow in aorta and branching arteries. It is this blood pressure pulsatility that is thought to have damaging effects on sensitive target organs as well as on vascular function, and to contribute to the vicious cycle of arterial remodeling.

High blood pressure pulsatility leads to increased mechanical vascular wall stress. With high central PP, the amplitude in which the arterial wall expands and contracts with each consecutive heartbeat is increased. This leads to higher stretch on elastic and collagen fibers in the arterial wall and this in turn may contribute to material fatigue, fracture, and degradation. Additionally, cyclic stretching of VSMC has been demonstrated to stimulate phenotype switching and arterial remodeling (Williams, 1998). Secondly, pathological blood pressure pulsatility adversely affects endothelial function since structure and function of the endothelium are modulated by hemodynamic forces (Gimbrone and García-Cardeña, 2012). In hypertensive patients, a high pulse-pressure is associated with endothelial dysfunction, which can be measured as the vasodilator response to acetylcholine (Ceravolo et al., 2003). In the normal situation, a laminar blood flow pattern and cyclic shear stress maintain proper endothelial function such as: NO-mediated regulation of vascular tone, maintaining a non-thrombotic and non-inflammatory state, preserving ECM metabolism, and regulating vascular permeability (Vita and Mitchell, 2003; Gimbrone and García-Cardeña, 2012). In arteries with remodeling, blood flow becomes increasingly oscillatory with peaked systolic flows as well as stasis and even flow reversal during diastole (Domanski et al., 1999; Mitchell, 2004). The ensuing turbulent flow and locally altered shear stress patterns cause endothelial dysfunction, which is characterized by impaired NO synthesis and upregulation of pro-inflammatory and pro-atherogenic factors, increased oxidative stress, as well as vasoconstriction (Keulenaer et al., 1998; Blackman et al., 2002; Gimbrone and García-Cardeña, 2012). In addition, altered flow and increased pressure pulsatility have been shown to activate the endothelium and induce production of osteogenic factors such as BMP-2 and BMP-4 (Qiu and Tarbell, 2000; Sorescu et al., 2003; Boström et al., 2011). Indeed, BMP-2 transgenic apoE^−/−^ mice display increased calcification of atheromatous lesions, whereas MGP transgenic apoE^−/−^ mice have less atherosclerotic mineralization, suggesting a key role for MGP in suppressing BMP-2-induced vascular mineralization (Nakagawa et al., 2010; Yao et al., 2010).

Arterial stiffness and endothelial function not only stimulate the development of atherosclerotic plaques but also further promotes arterial media remodeling. In this way, arterial stiffness may explain the interrelationship of arteriosclerosis and atherosclerosis.

Finally, the pathological hemodynamic patterns due to arterial stiffness lead to damage of susceptible organs such as kidneys, brain, and heart. It has been established that arterial stiffness and chronic kidney disease are closely interrelated (Safar et al., 2004). Patients with primary kidney disease have accelerated arterial remodeling and calcification due to altered homeostasis of calcium and phosphate, high degrees of inflammation and oxidative stress, uremia, altered cholesterol metabolism, and an activated renin-angiotensin system (RAS) (Safar et al., 2004). Conversely, increased arterial stiffness and pressure pulsatility induce renal damage (Verhave et al., 2005; Ford et al., 2010; Briet et al., 2011; Chen et al., 2011). Blood pressure pulsatility has been put forward to be able to cause renal damage. Although kidneys are normally protected against high blood pressure by an effective autoregulation, abnormal blood pressure pulsatility has been shown to blunt the renal myogenic response (Bidani and Griffin, 2004; Bidani et al., 2009; Hultström, 2012), exposing the vulnerable glomerular microcirculation to damaging pressure oscillations (Safar et al., 2012).

### Calcification as cardiovascular risk factor and possible therapeutic target

In PXE, PXE-like syndrome as well as in Keutel's syndrome, arterial calcification is an important feature of the clinical phenotype. Besides these, arterial calcification is also observed in more common disorders such as diabetes, hyperparathyroidism, and chronic kidney disease as well as in vascular aging. In addition, vascular calcification may be induced by drugs that adversely affect the regulatory balance between factors inducing or inhibiting calcification. For instance, chronic treatment with vitamin K-antagonists (such as warfarin) is associated with peripheral artery calcification (Rennenberg et al., 2010). Calcification occurs in both arteriosclerosis and atherosclerosis. Aortic medial calcification has been demonstrated to contribute to arterial stiffness in different populations (Odink et al., 2008; Cecelja et al., 2011; Sekikawa et al., 2012). Moreover, the presence of aortic calcification is predictive of coronary artery disease (Jang et al., 2012). Calcification of coronary arteries predominantly reflects atherosclerosis and can be measured and quantified by computed tomography (CT) using the calcium-score. The calcium score (expressed as Agatston units) has been used as a sensitive tool for risk stratification and decision-making regarding coronary revascularization and diagnostic angiography. A negative calcium score indicates that the presence of atherosclerotic plaque is very unlikely, whereas a high calcium score is associated with significant cardiovascular risk (Budoff et al., 2006). The importance of calcification with respect to cardiovascular outcome is further stressed by the fact that rapid annual progression of the calcium score is independently associated with outcome (Raggi et al., 2004). For this reason, the calcification process may become an important therapeutic target. The challenge is that an intervention should be aimed at a modifiable factor in the pathophysiological process. As can be learned from PXE, PXE-like syndrome and Keutel's syndrome, MGP and the vitamin K cycle are among the most important known regulators of calcification and VSMC phenotype switching. As described above, MGP requires vitamin K mediated carboxylation to be biologically active. Therefore, treatment with vitamin K would theoretically inhibit or possibly reverse arterial calcification and slow down the development of arterial stiffness. Indeed, our group demonstrated that calcification could be reversed in rats that had extensive calcification due to warfarin treatment, by subsequently administering vitamin K (Schurgers et al., 2007). In humans, the 3-year daily supplementation of 500 mcg vitamin K on top of a multi-vitamin resulted in hold on progression of vascular calcification (Shea et al., 2009) In the observational Rotterdam study, high dietary intake of vitamin K was associated with better cardiovascular outcome and reduced coronary artery calcification (Geleijnse et al., 2004; Gast et al., 2009). Also, in post-menopausal women, treatment with vitamin K resulted in improved markers of vascular stiffness (Braam et al., 2003). Furthermore, a recent study by Westenfeld et al. showed that vitamin K2 supplementation reduced plasma levels of inactive, undercarboxylated MGP (Westenfeld et al., 2012). Since vitamin K has no reported adverse side effects, it might be a promising treatment for calcification. Clinical trials investigating the effects of vitamin K supplementation on calcification and arterial remodeling are currently in progress.

### Arterial remodeling as potential therapeutic target

In addition to calcification, other pathophysiological pathways of arterial remodeling such as arterial stiffening, fibrosis, or elastin degradation may also be potential candidates for intervention. However, finding suitable, modifiable candidates has proven to be a challenge. Although most existing antihypertensive drugs may reduce arterial stiffness to some extent, it is difficult to determine whether this effect is mainly due to blood pressure reduction or represents a true effect on ECM remodeling (Boutouyrie et al., 2011). Since the RAS plays an important pro-fibrotic role in arterial remodeling it has been suggested that beneficial effects of RAS antagonists are (partly) due to their anti-fibrotic action, independent of their effects on blood pressure. Indeed, Tropeano et al. showed that treatment with 8 mg perindopril was associated with lower carotid stiffness independently of the effects on blood pressure, whereas a dose of 4 mg did not have such an effect (Tropeano et al., 2006). Similar blood-pressure-independent de-stiffening effects have been reported for selective aldosterone antagonists such as eplerenone (White et al., 2003), supporting possible effects of RAS system inhibition on ECM remodeling. Especially in diabetes, advanced glycation end-products (AGE) contribute to arterial stiffness by creating cross-links between elastic and collagen fibers. Therefore, the AGE crosslink-breaker alagebrium has received attention as potential de-stiffening drug (Zieman et al., 2007). This α-Aminoguadine compound improved aortic stiffness and improved peripheral arterial endothelial function in hypertensive patients, independently of blood pressure (Kass et al., 2001; Zieman et al., 2007). However, further research is required to properly assess the effects and safety of this class of drugs.

## Conclusion and future perspectives

Studying genetic diseases such as PXE, PXE-like syndrome, Keutel's syndrome and Marfan's disease increase our knowledge about pathophysiological mechanisms underlying arterial remodeling (summarized in Figures 2 and 3). Single gene defects of these specific diseases affect major regulatory pathways such as VSMC phenotype switching, matrix degradation, and calcification that are also involved in common cardiovascular disease and aging. Lessons learned from PXE, PXE-like syndrome and Keutel's syndrome have given attention to the major calcification regulatory protein MGP and has provided a possible new target for intervention. In this way, the continued study of these relatively rare genetic diseases may ultimately provide us with potential new targets for therapeutic intervention above and beyond traditional cardiovascular risk management and treatment of risk factors. Conceivably, since VSMC phenotype switching has such an important regulatory role in arterial remodeling, specifically targeting the direction of VSMC phenotype switching may prove to be promising. Ultimately, these novel concepts learned from studying specific genetic diseases can be applied to general cardiovascular medicine.

### Conflict of interest statement

The authors declare that the research was conducted in the absence of any commercial or financial relationships that could be construed as a potential conflict of interest.
